# Host cholesterol influences the activity of sterol biosynthesis inhibitors in *Leishmania amazonensis*

**DOI:** 10.1590/0074-02760220407

**Published:** 2022-04-04

**Authors:** Valter Viana Andrade-Neto, Pedro Paulo de Abreu Manso, Miria Gomes Pereira, Nuccia Nicole Theodoro de Cicco, Georgia Corrêa Atella, Marcelo Pelajo-Machado, Rubem Figueiredo Sadok Menna-Barreto, Eduardo Caio Torres-Santos

**Affiliations:** 1 Fundação Oswaldo Cruz-Fiocruz, Instituto Oswaldo Cruz, Laboratório de Bioquímica de Tripanossomatídeos, Rio de Janeiro, RJ, Brasil; 2 Fundação Oswaldo Cruz-Fiocruz, Instituto Oswaldo Cruz, Laboratório de Patologia, Rio de Janeiro, RJ, Brasil; 3 Universidade Federal do Rio de Janeiro, Instituto de Biofísica Carlos Chagas Filho, Laboratório de Ultraestrutura Celular Hertha Meyer, Rio de Janeiro, RJ, Brasil; 4 Universidade Federal do Rio de Janeiro, Instituto de Bioquímica Médica, Rio de Janeiro, RJ, Brasil; 5 Fundação Oswaldo Cruz-Fiocruz, Instituto Oswaldo Cruz, Laboratório de Biologia Celular, Rio de Janeiro, RJ, Brasil

**Keywords:** leishmaniasis, low-density lipoprotein, cholesterol, sterol biosynthesis inhibitors

## Abstract

A significant percentage of exogenous cholesterol was found in promastigotes and amastigotes of all studied species of *Leishmania*, suggesting a biological role for this molecule. Previous studies have shown that promastigotes of *Leishmania* uptake more low-density lipoprotein (LDL) particles under pharmacological pressure and are more susceptible to ergosterol inhibition in the absence of exogenous sources of cholesterol. This work shows that the host’s LDL is available to intracellular amastigotes and that the absence of exogenous cholesterol enhances the potency of sterol biosynthesis inhibitors in infected macrophages. A complete understanding of cholesterol transport to the parasitophorous vacuole can guide the development of a new drug class to be used in combination with sterol biosynthesis inhibitors for the treatment of leishmaniases.

**Leishmaniasis**

Leishmaniases are caused by parasites of the genus *Leishmania*; they are unicellular eukaryotes that belong to the family Trypanosomatidae. The disease presents with several clinical manifestations, such as cutaneous, mucocutaneous, and visceral manifestations, and it occurs in the old and new worlds. More than 90% of visceral leishmaniasis cases worldwide have occurred in seven countries (Brazil, Ethiopia, India, Kenya, Somalia, South Sudan, and Sudan). In addition, Brazil is among the countries where more than 90% of cases of cutaneous leishmaniasis have been reported worldwide.^1^ ^,^ ^2^ Since the 1940s, two commercially available formulations of pentavalent antimonials have been available for the treatment of leishmaniasis: *N*-methylglucamine antimoniate (meglumine antimoniate) (Glucantime^®^) and sodium stibogluconate (Pentostam^®^). Miltefosine, the only licensed oral drug for the treatment of leishmaniasis, has been employed for cutaneous and visceral leishmaniasis in some countries, such as India, Germany, and Colombia;^1^ ^,^ ^2^ ^,^ ^3^ however, resistant cases have been reported, with increased failure in the treatment of visceral leishmaniasis.^3^ Other drugs, such as pentamidine, amphotericin B, and paromomycin, are used in some circumstances, such as in cases of resistance to first-line treatment, even though they have significant toxic effects.^1^ ^,^ ^2^

**Sterol biosynthesis in *Leishmania* spp.**

Sterols are structural lipids and are present in the membranes of most eukaryotic cells. Cholesterol is the essential mammalian cell sterol, but plants, fungi, and some protozoa synthesise other closely related sterols instead of cholesterol using the same synthetic pathway up to the 2,3-squalene epoxide step.^4^ *Leishmania* spp. produce ergosterol and other 24-alkylated sterols (ergostane-based sterols) that are considered essential for both promastigote and amastigote forms.^5^ ^,^ ^6^ ^,^ ^7^

The sterol biosynthetic pathway of *Leishmania* spp. and *Trypanosoma cruzi* has been deduced from studies with [^14^C]-acetate and [^14^C]-mevalonate and from the identification of intermediate sterols that accumulate after treatment with various sterol biosynthesis inhibitors.^5^ ^,^ ^8^ The discovery of these intermediates suggested that sterol biosynthesis in *Leishmania* spp. occurs similarly to that in fungi, offering an opportunity for the development of targeted chemotherapy in the sterol biosynthetic pathway.^4^ ^,^ ^9^ In fact, the inhibition of several enzymes of this pathway, such as HMG-CoA reductase, squalene epoxidase, lanosterol C-14 demethylase, and C-24 methyltransferase, by statins (simvastatin, lovastatin, and atorvastatin), allylamines (terbinafine), azoles (ketoconazole, miconazole, posaconazole, and itraconazole) and azasterols causes the death of *Leishmania* spp.^5^ ^,^ ^10^ ^,^ ^11^ ^,^ ^12^ ^,^ ^13^ ^,^ ^14^ Therefore, the obvious conclusion is that some of these drugs, which have already been successfully used in clinics against fungal infections, could be alternatives for the treatment of leishmaniases.^5^ ^,^ ^9^

**Uptake of exogenous cholesterol**

Although *Leishmania* spp. synthesise ergostane-derived sterols endogenously, they also obtain exogenous cholesterol, mainly through a low-density lipoprotein (LDL) receptor that is conserved in several trypanosomatids. This receptor is located in the flagellar pocket, and studies have shown that the cholesterol obtained by this parasite comes from the uptake and metabolism of LDL, which mainly contains cholesterol and cholesterol esters.^15^

*Leishmania amazonensis* promastigotes, under pharmacological pressure from sterol biosynthesis inhibitors (ketoconazole, miconazole, and simvastatin), endocytose more LDL from the culture medium. They are also more sensitive to these inhibitors when they are deprived of exogenous cholesterol sources, suggesting that cholesterol uptake may play a compensatory role when the ergosterol biosynthetic pathway is inhibited.^16^ ^,^ ^17^ Disturbance of sterol biosynthesis by itraconazole and ketoconazole was also analysed in *L. mexicana* amastigotes, and this life cycle stage was shown to have greater sensitivity to these molecules. Treatment of amastigotes with itraconazole and ketoconazole was also shown to cause increased amounts of exogenous cholesterol, as seen in the untreated promastigote forms.^18^

**Leishmaniasis and cholesterol metabolism**

Lipid alterations in plasma have been demonstrated in some infectious diseases, and cholesterol has been studied in many parasitic infections.^19^ Specifically, in leishmaniasis, two study approaches are used globally: the correlation of systemic cholesterol homeostasis with the disease and the relationship of host cell cholesterol with the infection. The first approach is derived from observations that patients with visceral leishmaniasis have hypocholesterolemia. The second approach is derived from the importance of lipid rafts for the entry of the parasite into the macrophage and the presentation of antigens. Patients with visceral leishmaniasis have a decrease in serum LDL-cholesterol, leading to hypocholesterolemia, which is mainly evident in this form of the disease.^20^ ^,^ ^21^ The two study approaches overlap in some respects but differ essentially in that macrophages per se are not responsible for cholesterol homeostasis, a role played mainly by hepatocytes.

Ghosh and colleagues^22^ showed that hypercholesterolemia offers protection against infection caused by *L. donovani*, whereas hypocholesterolemia makes mice more susceptible to infection with the parasite.^22^ Recent works have shown that the hypocholesterolemia observed in *L. donovani* infection is due to the release of metalloprotease GP63 by the parasites infecting Kupffer cells in the liver. GP63 cleaves dicer (endonuclease RNAse III) in hepatocytes, reducing the expression of miR122 and leading to reduced production of cholesterol. miR122 is a posttranscriptional regulator (miRNA) that is abundantly expressed in the liver and modulates various functions. It comprises more than 70% of the miRNA in the liver and is mainly responsible for homeostasis and lipid metabolism. The downstream events of GP63 release affect cholesterol homeostasis, leading to progression of the infection due to failure in antigen presentation.^23^ ^,^ ^24^ However, the mechanisms of transport of GP63 from the parasitophorous vacuole of Kupffer cells to hepatocytes and the expression of GP63 by amastigotes remain to be elucidated. Corroborating these data, parallel studies have shown that lipoproteins can modulate the cellular immune response in leishmaniasis.^25^ Other studies have shown that macrophages infected with *L. donovani* have significantly decreased membrane cholesterol. The administration of cholesterol in infected hamsters offers substantial protection, showing the importance of cholesterol in the macrophage membrane for correct antigen presentation and immune system activation.^26^ ^,^ ^27^

On the other hand, the results published by Fernandes and colleagues^28^ revealed that infection by *L. major* significantly increases the migration of inflammatory cells to atherosclerotic lesions and promotes atherogenesis. The process of cholesterolemia accompanies this increase. These effects of *L. major* primary infection are a consequence of the stimulation of the immune system, specifically stimulation of the inflammatory components of atherosclerosis, which are activated by parasites in macrophages.^28^ There are some controversial results regarding the modulation of host cholesterol homeostasis, but there seems to be no doubt that *Leishmania* infection alters host cell cholesterol metabolism.

**Availability of LDL particles for intracellular amastigotes**

LDL is the primary extracellular carrier of cholesterol, playing an essential physiological role in cell function and the regulation of metabolic pathways. Macrophages have LDL receptors that endocytose these particles under normal conditions. Under pathological conditions, such as hyperlipidemia, genetic disorders, and oxidative stress, specific components of LDL are oxidised, increasing its uptake.^29^

Semine and colleagues^30^ showed cholesterol trafficking in macrophages infected by *L. mexicana*. Infected macrophages increase LDL-cholesterol uptake and simultaneously decrease the level of NPC intracellular cholesterol transporter 1 (NPC1), which is responsible for late lysosomal cholesterol efflux, leading to retention of exogenous cholesterol in the parasitophorous vacuole (PV). Cholesterol concentrates around the parasites, forming a coating directly proportional to the number of parasites.^30^

Furthermore, the parasites take up cholesterol from the host cell membrane. Cholesterol sequestration occurs soon after the onset of infection; this helps explain the cholesterol-dependent process in macrophages that influences the function of the immune response, such as antigen presentation.^30^ This increase in cholesterol retention in the PV may be related to the decrease in cholesterol in the macrophage membrane, decreasing antigen presentation and contributing to disease progression.

Other works have demonstrated that infected macrophages alter their cholesterol metabolism by increasing expression of pathway enzymes, increasing LDL receptors, and decreasing ABC transporters that efflux cholesterol, thus leading to an accumulation of cholesterol within these cells.^31^ ^,^ ^32^ The accumulated cholesterol may be used in some way by the parasite, since the presence of cholesterol in amastigotes has already been demonstrated.^18^ ^,^ ^33^

Since the central cell affected in *L. amazonensis* infection is the macrophage, and it has LDL receptors, we studied whether amastigotes use LDL particles. To assess the availability of LDL to intracellular amastigotes, peritoneal macrophages were infected with *L. amazonensis*-GFP and incubated with LDL-Alexa 594 or infected with *L. amazonensis* and incubated with LDL-gold.

First, for the confocal microscopy experiments, peritoneal macrophages from Swiss mice (1.0 x 10^6^/mL) were plated and adhered to Lab-teks (Thermo-Fisher) for 1 hour and infected with 3.0 x 10^6^/mL metacyclic promastigotes of *L. amazonensis*-GFP (MHOM/BR/77/Josefa strain) for 72 h at 37ºC. Macrophages were washed and incubated with serum-free medium 24 h before incubation with LDL-labeled Alexa Fluor^®^ 594 AcLDL (Invitrogen, Life Technologies Corporation). LDL particles (red) can be visualised very close to the intracellular amastigotes, probably inside the PV (Fig. 1). The PV has proteins that can transport molecules from or to the cytoplasm. In addition to this active transport, intracellular vesicles, such as those formed by LDL endocytosis, can fuse with PV, discharging its intravesicular content.

**Fig. 1:**
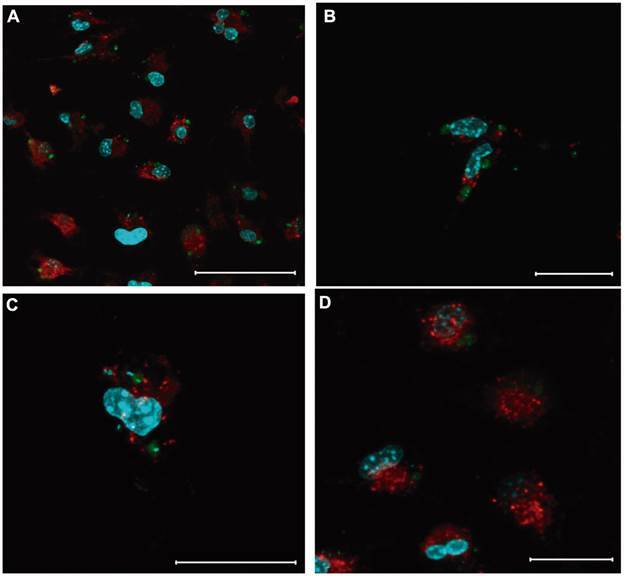
distribution of low-density lipoprotein (LDL) particles in macrophages infected with *Leishmania amazonensis*-GFP. Peritoneal macrophages infected with *L. amazonensis*-GFP were incubated with LDL-Alexa 594 particles for 30 (A and B) or 150 min (C - D) at concentrations of 0.125 (A and C) and 0.5 µg/mL (B and D). After this period, the cells were washed three times in phosphate buffered saline (PBS), fixed with 4% formaldehyde for 10 min and incubated with DAPI for 20 min. Slides were mounted using Prolong^®^ Gold Antifade Mounting Fluid (Invitrogen, Life Technologies Corporation) and analysed under a Zeiss LSM 510 META confocal microscope. Green: *L. amazonensis* amastigotes; red: LDL particles; blue: nuclei/kinetoplasts (DAPI). Bars: A = 50 µm; B-D = 20 µm.

For the transmission electron microscopy experiments, peritoneal macrophages from Swiss mice (1.0 x 10^6^/mL) were plated, adhered to small Petri dishes for 1 hour, and infected with 3.0 x 10^6^/mL metacyclic promastigotes of *L. amazonensis* (strain MHOM/BR/77/LTB0016) for 3 h. After this period, the plates were washed with serum-free medium and incubated for 72 h at 37ºC. Four hours before the end of the final incubation period, the plates were incubated with colloidal gold-labeled LDL^34^ at a 1:10 dilution. After this period, infected and uninfected macrophages were washed with phosphate buffered saline (PBS) and fixed for 40 min in a 2.5% glutaraldehyde solution diluted in 0.1 M sodium cacodylate buffer, pH 7.4.^35^ Ultrathin sections were collected, stained with uranyl acetate and lead citrate, and examined in a Jeol 1011 transmission electron microscope (Tokyo, Japan) at Plataforma de Microscopia Eletrônica at Fiocruz. LDL purification was performed as described by Poumay and Ronveaux,^36^ with some modifications.

The confocal microscopy results were corroborated by transmission electron microscopy. Fig. 2A-B shows the distribution of LDL particles in uninfected macrophages. LDL-gold particles (dark spots) were endocytosed and stored in endocytic vacuoles to be processed and used by the host cell. In Fig. 2C-D, macrophages infected with *L. amazonensis* were incubated with LDL-gold particles. The endocytic vacuoles containing the LDL particles fused with the PV. We also observed the presence of free LDL particles inside the PV, even at very close distances to the amastigotes, suggesting adhesion to the parasite membrane. These images demonstrate that intracellular amastigotes have access to LDL and, therefore, may be obtaining their content, mainly cholesterol. The parasite may use the cholesterol of the LDL particles inside the PV, since the presence of cholesterol in amastigotes has already been demonstrated.^18^ ^,^ ^33^

**Fig. 2:**
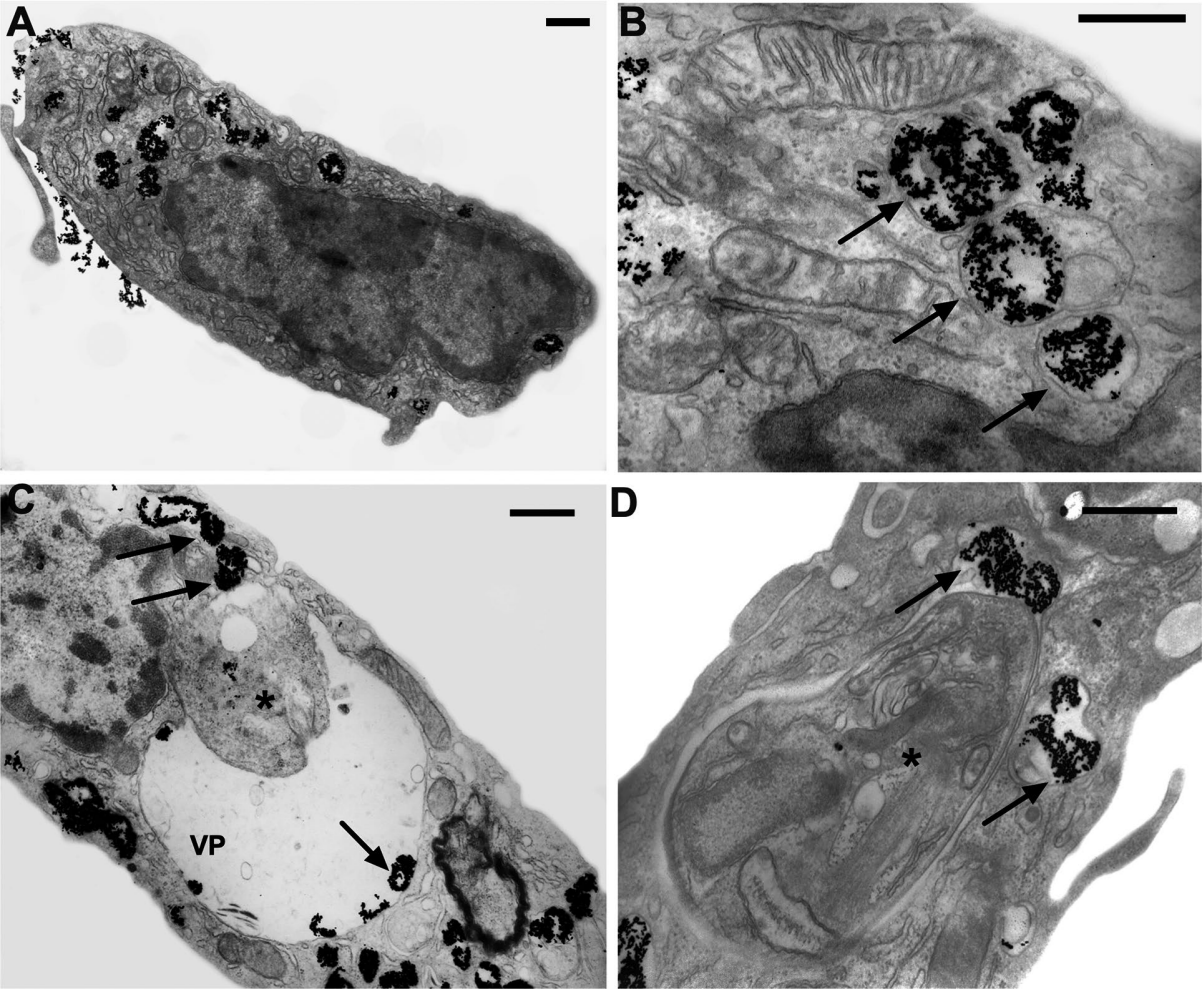
availability of low-density lipoprotein (LDL)-gold to intracellular amastigotes. Peritoneal macrophages infected or not with *Leishmania amazonensis* were incubated with LDL-gold (10 nm) for 4 h and analysed by transmission electron microscopy. A and B: uninfected macrophages; C and D: infected macrophages. Arrows: LDL particles; asterisks: *L. amazonensis* amastigotes; VP: parasitophorous vacuole. The asterisk represents the amastigotes within the parasitophorous vacuole. Bars = 0.5 µm.

**Influence of exogenous cholesterol on the leishmanicidal activity on intracellular amastigotes**

After observing that intracellular amastigotes access LDL endocytosed by macrophages, we evaluated the effect of the absence of exogenous cholesterol sources on the leishmanicidal activity of ketoconazole and miconazole. Murine peritoneal macrophages (1.0 x 10^6^ cells/mL) were infected with 3 x 10^6^ promastigotes/mL of *L. amazonensis* for 3 hours and incubated in RPMI medium with cholesterol-free Nutridoma^®^ (Roche) or foetal bovine serum (FBS) in the absence or presence of miconazole or ketoconazole (0-16 µM) for 72 h. After incubation, the slides were stained with fast panoptic, and the infection index was determined by counting under an optical microscope. The infection index was calculated using the formula: % infected macrophages x nº of amastigotes/nº total of macrophages.

The parasites became more sensitive to the leishmanicidal activity of ketoconazole (Fig. 3A) and miconazole (Fig. 3B) when infected macrophages were cultured in Nutridoma compared to FBS. Ketoconazole had an EC_50_ of 4.7 µM (95% CI: 4.2-5.4 µM) in the presence of FBS, while Nutridoma had an EC_50_ of 1.5 µM (95% CI: 1.1-2.0 µM). Miconazole had an EC_50_ of 2.9 µM (95% CI: 2.3-3.6 µM) with FBS and 0.77 µM (95% CI: 0.68-0.85 µM) with Nutridoma. The results suggest that the absence of exogenous cholesterol can influence the activity of ergosterol biosynthesis inhibitors. Intracellular amastigotes were more sensitive to ketoconazole and miconazole when incubated in cholesterol-free medium, suggesting that even in the intracellular environment, *L. amazonensis* uses cholesterol to replace ergosterol in an attempt to maintain its membrane properties.

**Fig. 3:**
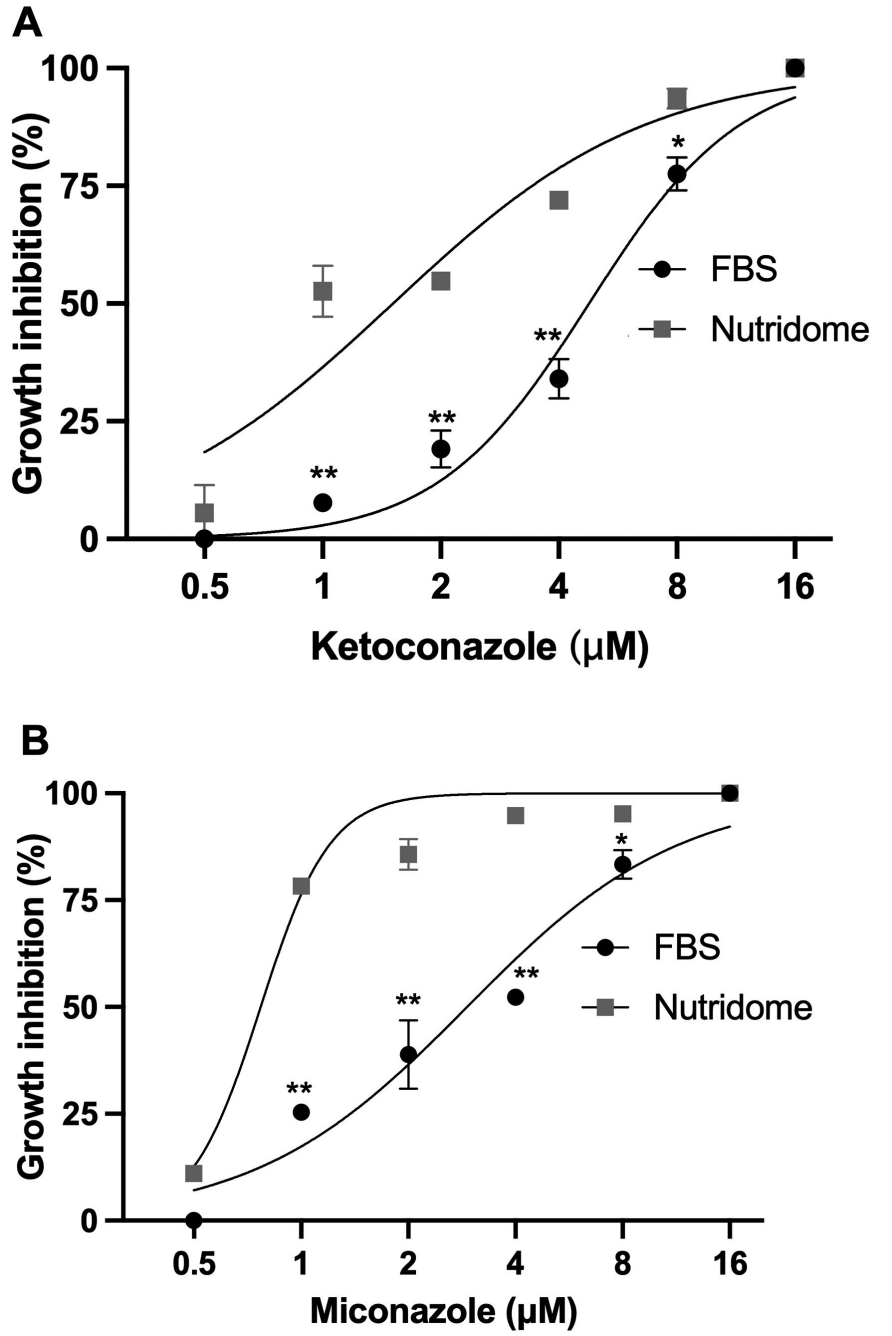
effect of the absence of exogenous cholesterol on the leishmanicidal activity of ketoconazole and miconazole. Peritoneal macrophages infected with *Leishmania amazonensis* were incubated in RPMI culture medium supplemented with foetal bovine serum (FBS) or cholesterol-free Nutridoma. Cultures remained untreated or were treated with ketoconazole (A) or miconazole (B) for 72 h. The experiments were carried out in triplicate, n = 3. The graphs are representative of an experiment with the standard deviation value. The graphs and IC_50_ values were obtained using the GraphPad Prism 7 program. Student’s *t test*, *p < 0.5, **p > 0.001.

**Concluding remarks**

In conclusion, the data presented here suggest that intracellular amastigotes access the host’s LDL and that the availability of this pool of cholesterol can be used by the parasite to overcome the effect of sterol biosynthesis inhibitors. A complete understanding of this phenomenon can guide the development of a new drug class to be used in combination with sterol biosynthesis inhibitors for the treatment of leishmaniases.
